# Effects of *STIP1* and *GLCCI1* polymorphisms on the risk of childhood asthma and inhaled corticosteroid response in Chinese asthmatic children

**DOI:** 10.1186/s12890-020-01332-2

**Published:** 2020-11-18

**Authors:** Juan Huang, Xiaolei Hu, Xiangrong Zheng, Jian Kuang, Chentao Liu, Xia Wang, Yongjun Tang

**Affiliations:** 1 Department of Pediatric, Xiangya Hospital, Central South University, Changsha, Hunan China; 2 Department of Pediatric, The First Hospital of Changsha, Changsha, Hunan China; 3 National Institution of Drug Clinical Trial, Xiangya Hospital, Central South University, Changsha, Hunan China

**Keywords:** Childhood asthma, *STIP1*, *GLCCI1*, Polymorphism, Pulmonary function

## Abstract

**Background:**

Asthma is a common chronic lung disease in children. We aimed to determine the associations between *stress-induced phosphoprotein 1 (STIP1)* and *glucocorticoid-induced transcript 1 (GLCCI1)* polymorphisms and susceptibility of childhood asthma and inhaled corticosteroid (ICS) response in children.

**Methods:**

A total of 263 Chinese Han asthmatic children were recruited from the Xiangya Hospital, Central South University. Pulmonary function tests were performed before the treatment and 3 months after the treatment. One hundred fifty non-asthmatic children were recruited. Each participant’s DNA was extracted from the peripheral blood and Method of MassARRAY was used to genotype the single-nucleotide polymorphisms (SNPs).

**Results:**

*STIP1* rs2236647 wild-type homozygote (CC) was associated with increased asthma risk of children (OR = 1.858, 95% CI:1.205–2.864), but not associated with the ICS response. *GLCCI1* rs37969, rs37972 and rs37973 polymorphisms were not associated with the risk of childhood asthma. However, rs37969 mutant genotypes (TT/GT) were significantly associated with less improvement in PD20 (*p* = 0.028). We also found significant associations between rs37969, rs37972 and rs37973 mutant genotypes and less improvement in maximal midexpiratory flow (MMEF) after ICS treatment for 3 months (*p* = 0.036, *p* = 0.010 and *p* = 0.003, respectively).

**Conclusions:**

*STIP1* rs2236647 was associated with asthma risk of children and *GLCCI1* rs37969 mutant genotypes were associated with less improvement in airway hyper-responsiveness. *GLCCI1* rs37969, rs37972 and rs37973 polymorphisms might be associated with pulmonary function in childhood asthma patients after ICS treatment.

**Supplementary Information:**

The online version contains supplementary material available at 10.1186/s12890-020-01332-2.

## Background

Asthma is one of the most common chronic lung diseases in children and adults. Approximately 358 million individuals suffer from asthma in the world [1]. The average global prevalence of adult asthma is 4.3%, up to 21.0% in Australia [2]. Simultaneously, the global prevalence of asthma in children aged 6 to 7 years and in those aged 13 to 14 years are respectively 11.6 and 13.7% [3]. In developed countries, the prevalence increased more obviously in the past few years. Meanwhile, the clinical control of asthma is not promising. Uncontrolled asthma accounts for 53.4% in Asian pediatric asthma and 45% in European adult asthma [4, 5]. In China, only 28.7% of patients achieved complete asthma control [6]. Asthma is an important contributor to the burden of families and society around the world. Therefore, reducing the prevalence of asthma and improving asthma control will significantly decrease the global medical burden and meaningfully promote the development of global health care.

The current perspective is that the drug response of asthma in children and adults are closely associated with genetic factors. Studies have shown that genetic factors contribute about 70% of the variability in inhaled corticosteroid (ICS) response [7, 8]. There are more than 1000 candidate genes had been discovered in the genome-wide association studies (GWAS) [9], and approximately 50 genes have been replicated identified [10].

Many of the replicated genes were involved in the steroid molecular pathway and one of the important genes in the steroid molecular pathway is *stress-induced phosphoprotein 1 (STIP1)*. *STIP1* contains 14 exons and encodes heat shock organizing proteins (hops) that participate in the activation of glucocorticoid receptor (GR). GR is usually inactive and activated with the help of hop-hsp90 complex [11, 12]. Then, the GR-glucocorticoid complex can suppress airway inflammation, inhibit the activation of inflammatory genes, and regulate the activity and transcription of airway remodeling genes. In short, the *STIP1* gene can affect the binding process of glucocorticoid (GC) and GR, thereby affecting the efficacy of GC. 3 *STIP1* single-nucleotide polymorphisms (SNPs; rs4980524, rs6591838 and rs2236647) were found to be associated with ICS response in a white adult population [13] and another study found *STIP1* rs2236647 polymorphism was also associated with the risk of asthma in adult population of Arab descent [14]. Besides, no association was found between *STIP1* SNPs and change in FEV1 after ICS treatment in the study of childhood asthma in Korea and adult asthma in Japan [15].

*Glucocorticoid-induced transcript 1(GLCCI1)* also plays a key role in steroid biology and involved essentially in asthma signaling [16]. *GLCCI1* contains 8 exons and encodes glucocorticoid-induced transcript 1 that promotes the anti-inflammatory effects of glucocorticoids [17]. A GWAS study indicated that *GLCCI1* rs37972 polymorphism was associated with ICS response in white childhood asthma patients and replicated their findings in three adult clinical trials and a Network childhood asthma trial (Data from the database of Genotypes and Phenotypes, dbGaP) [16]. However, the result was not repeated in north European asthmatic children [18]. *Hu C* et al. found that *GLCCI1* variations (rs37972, rs37973 and rs11976862 polymorphisms) were associated with ICS response and asthma susceptibility in Chinese adult [19]. A recent study indicated that *GLCCI1* variants (rs37972 and rs37973 polymorphisms) could serve as asthma risk biomarkers in a Tunisian adult population [20].

**Fig. 1:**
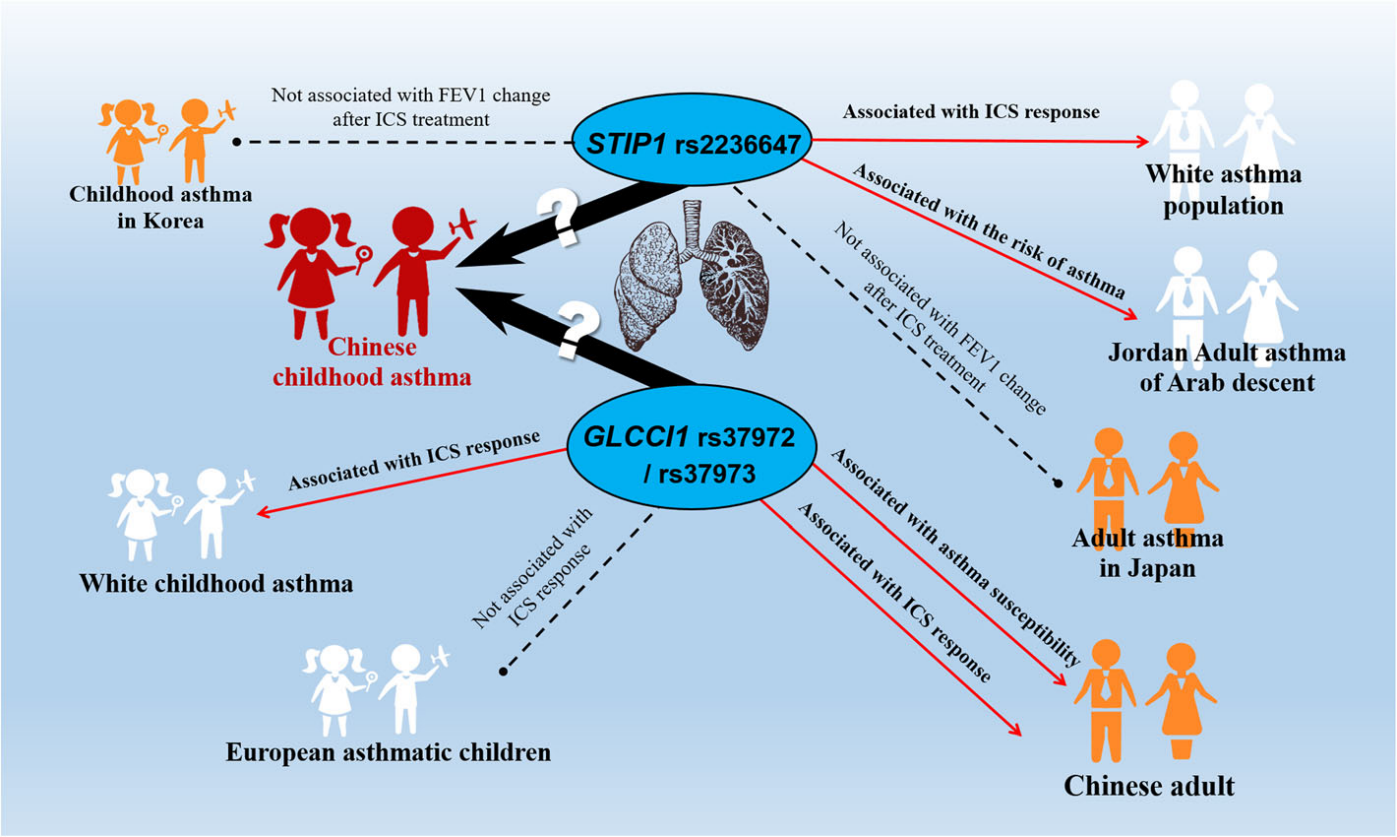
Current research status of *STIP1* rs2236647 and *GLCCI1* rs37972 /rs37973 polymorphisms in asthma of different populations. ICS: Inhaled corticosteroid

Figure 1 summarizes the advances of *STIP1* rs2236647 and *GLCCI1* rs37972 /rs37973 polymorphisms in asthma researches. And currently, the studies on the above two genes (*STIP1* and *GLCCI1*) and Chinese childhood asthma are still rarely reported. The aim of our study is to investigate the effects of *STIP1* and *GLCCI1* polymorphisms on the risk of childhood asthma and ICS response in Chinese asthmatic children.

## Methods

### Subjects

Two hundred sixty-three Chinese Han asthmatic children and 150 controls were recruited from the Xiangya Hospital of Central South University. All the asthmatic patients received ICS treatment for 3 months (inclusion and exclusion criteria of all cases are listed in Table 1). These subjects all come from Hunan, China.

**Table 1:** Inclusion criteria for enrollment in case-control study and the treatment trial

**Asthmatics inclusion criteria**	
1. Meets the 2017 GINA guidelines on the diagnostic criteria for asthma.	
2. No history of respiratory infections and systemic infections within 1 month.	
3. No history of hospitalization for asthma exacerbation within 1 month.	
4. No regular history of using ICS within 1 month.	
5. No following diseases: congenital lung malformations; airway obstruction or extraluminal oppression; congenital heart disease; active tuberculosis; bronchiectasis; severe systemic diseases.	
**Controls inclusion criteria**	
Without the following diseases: Bronchial asthma; Bronchiolitis; Allergic diseases such as eczema, allergic rhinitis and atopic dermatitis; Severe systemic diseases; Family history of allergic diseases.	

### Pulmonary function test

Pulmonary function was performed using the Jaeger Masterscope spirometry system (Jaeger, Wuerzburg, Germany). All asthmatic children over 6 years of age had pulmonary function measured at their first visit and after 3 months of ICS treatment. Forced expiratory volume in 1 s (FEV1)/pre, FEV1/Forced vital capacity (FVC), Peak expiratory flow (PEF)/pre, Forced expiratory flow (FEF) 25/pre, FEF 75/pre and Maximal midexpiratory flow (MMEF)/pre were used to evaluate the pulmonary function. The provocative dose of methacholine causing a 20% drop in FEV1 (PD20) was used to represent airway hyper-responsiveness.

### Selection of SNPs

In this study, 4 SNPs in two genes (rs2236647 in *STIP1*; rs37969, rs37972, and rs37973 in *GLCCI1*) were investigated for their associations with asthma in children of China. The studied genes were selected based on their known biological functions in lung and their role in ICS response. These SNPs were selected from previous studies and database information (NCBI, https://www.ncbi.nlm.nih.gov/pubmed).

### Genotyping

Genotyping was performed using the iPlex MassARRAY genotyping platform (Sequenom, Inc., San Diego, CA). DNA was extracted from 1 mL of the collected blood using a DNA extraction kit (SQ Blood DNA KitII, Omega, USA). The primers were designed by AssayDesigner3.1 (Details are listed in Additional file 1: Table S1).

### Statistical methods

Statistical analyses were performed using PLINK 1.07 and SPSS v.18.0 (SPSS Inc., Tokyo, Japan). *p* > 0.05 was considered to be consistent with Hardy-Weinberg equilibrium (HWE). The chi-squared test was used to calculate significant differences in allele and genotype frequencies between asthmatics and controls. Odds ratios (OR) and 95% confidence intervals (CI) for asthma susceptibility in relation to the SNPs were performed by logistic regression analysis. Multivariate logistic regression analysis was used to adjust for age and gender. Analysis of variance (ANOVA) test and t-test were used to determine the influence of genotype on spirometry. *p* ≤ 0.05 were considered significant.

## Results

### Subject characteristics

We recruited 263 asthmatics (188 males, 75 females, mean age 8.18 ± 2.73 years) and 150 non-asthmatic controls (97 males, 53 females; mean age 8.20 ± 2.28 years) in our study. There was no difference between 2 groups in age, gender and smoking exposure (*p-*value: 0.725, 0.150 and 0.135, respectively). All patients received ICS treatment. Two hundred nine patients were over 6 years of age and 54 were under 6 years of age. In the patients over 6 years of age, 134 completed the 3-months follow-up. Other patients did not have follow-up or substandard treatment. The detailed baseline demographics of subjects are listed in Table 2.

**Table 2:** Baseline demographics of subjects involved in the study

Characteristics	N (%)		***P***-value
	Asthma patients	Controls	
Total	263	150	
Age (year)			
< 6	54(20.53)	33(22.00)	0.725
≥ 6	209(79.47)	117(78.00)	
Gender			
Boys	188(71.48)	97(64.67)	0.150
Girls	75(28.52)	53(35.33)	
Smoking exposure			
Yes	154(58.56)	99(66.00)	0.135
No	109(41.44)	51(34.00)	
Allergies			
Yes	172(65.40)	0(0.00)	
No	91(34.60)	150(100.00)	
Family history of asthma			
Yes	89(33.84)	0(0.00)	
No	174(66.16)	150(100.00)	

### *STIP1* rs2236647 was associated with the risk of childhood asthma

All the SNPs involved in our study were in HWE (Additional file 1: Table S2). The allele and genotype frequencies of the 4 SNPs in asthmatics and controls were listed in Table 3. We found allele frequencies and genotype frequencies of *STIP1* rs2236647 in asthmatics and controls were significantly different (*p* = 0.008 and *p* = 0.018, respectively; Table 3). Children with *STIP1* rs2236647 CC genotype showed increased risk of asthma compared with the other two genotypes (*p* = 0.005, Table 3). After adjusting for age and gender, we found that rs2236647 CC genotype was still associated with increased the risk of childhood asthma (OR = 1.929; Table 4). However, similar associations were not found in rs37969, rs37972, and rs37973 polymorphisms (Table 3).

**Table 3:** The allele and genotype frequency of 4 SNPs in asthmatics and controls

Gene	SNP	Genotype / Allele	Cases (***n*** = 263)	Controls (***n*** = 150)	***P-***value/Corrected ***p-***value^**a**^	REC^**b**^ model ***p-***value/Corrected ***p-***value^**a**^	DOM^**c**^ model ***p-***value/Corrected ***p-***value^**a**^
*STIP1*	rs2236647	TT	38(14.44%)	29(19.33%)	**0.018**/0.072	0.195/0.780	**0.005/0.020**
		TC	113(42.97%)	78(52.00%)			
		CC	112(42.59%)	43(28.67%)			
		T	189(35.93%)	136(45.33%)	**0.008/0.032**		
		C	337(64.07%)	164(54.67%)			
*GLCCI1*	rs37969	TT	53(20.15%)	34(22.67%)	0.820/1.000	0.547/1.000	0.708/1.000
		GT	130(49.43%)	73(48.67%)			
		GG	80(30.42%)	43(28.67%)			
		T	236(44.87%)	141(47.00%)	0.554/1.000		
		G	290(55.13%)	159(53.00%)			
	rs37972	TT	47(17.88%)	28(18.67%)			
		TC	121(46.01%)	70(46.67%)	0.952/1.000	0.840/1.000	0.766/1.000
		CC	95(36.12%)	52(34.67%)			
		T	215(40.87%)	126(42.00%)	0.752/1.000		
		C	311(59.13%)	174(58.00%)			
	rs37973	GG	54(20.53%)	36(24.00%)	0.675/1.000	0.412/1.000	0.550/1.000
		GA	128(48.67%)	72(48.00%)			
		AA	81(30.80%)	42(28.00%)			
		G	236(44.87%)	144(48.00%)	0.385/1.000		
		A	290(55.13%)	156(52.00%)			

The values *p* ≤ 0.05 were in bold

^a^Corrected by Bonferroni multiple adjustment; ^b^REC means (AA + Aa) vs aa; ^c^DOM means AA vs (Aa + aa); “A” is the major allele and “a” is the minor allele

**Table 4:** Association (OR, 95% CI) between gene SNPs and childhood asthma susceptibility^a^

SNP		OR	OR corr ^**b**^
rs2236647	CC vs (TC + TT)	1.846(1.201–2.838) ^⁎⁎^	1.929(1.247–2.986) ^⁎⁎^

^a^Table only shows SNPs that are associated with asthma susceptibility through logistic regression analysis of alleles and different genotypes

^b^OR corr: the *p* value after adjusting age, gender and smoking exposure as covariates; OR: Odds ratio (reference group designated with an OR of 1.0)

^⁎^ *p* ≤ 0.05; ^⁎⁎^ *p* ≤ 0.01

### 4 candidate SNPs were not associated with baseline lung function measures

Baseline lung function of different genotypes is shown in Table 5 and we found four SNPs (rs2236647, rs37969, rs37972, and rs37973) were not associated with baseline lung function measures (FEV1/pre, FEV1/FVC, PEF/pre, FEF 25/pre FEF 75/pre and MMEF/pre; Table 5). We also did not observe significant associations between baseline PD20 and the 4 SNPs (Table 5).

**Table 5:** Baseline lung function of different genotypes

Gene	SNP	Allele	FEV1/pre (%)	FEV1/FVC	PEF/pre (%)	FEF 25/pre (%)	FEF 75/pre (%)	MMEF/pre (%)	PD20 (mg)
*STIP1*	rs2236647	CT + TT	93.18 ± 11.74	94.69 ± 9.64	88.73 ± 13.24	80.36 ± 17.67	56.78 ± 20.53	66.32 ± 18.95	0.76 ± 0.76
		CC	93.80 ± 14.78	95.27 ± 9.30	86.89 ± 16.33	83.25 ± 22.70	55.48 ± 21.67	66.18 ± 21.96	0.83 ± 0.86
*GLCCI1*	rs37969	GT + TT	93.93 ± 12.90	95.10 ± 10.15	88.07 ± 15.25	82.36 ± 20.82	57.3 ± 22.32	67.06 ± 20.89	0.84 ± 0.84
		GG	92.24 ± 12.86	94.44 ± 7.94	88.07 ± 12.47	79.29 ± 16.59	54.16 ± 17.32	64.53 ± 18.01	0.66 ± 0.69
	rs37972	CT + TT	92.97 ± 12.84	94.69 ± 10.46	87.33 ± 14.57	80.63 ± 20.28	56.17 ± 22.65	65.79 ± 21.01	0.74 ± 0.77
		CC	94.09 ± 13.00	95.23 ± 7.74	89.29 ± 14.15	82.65 ± 18.51	56.55 ± 17.81	67.05 ± 18.40	0.84 ± 0.84
	rs37973	AG + GG	93.82 ± 12.75	95.00 ± 10.01	87.53 ± 14.52	81.09 ± 19.6	57.38 ± 22.09	66.68 ± 20.35	0.79 ± 0.80
		AA	92.41 ± 13.24	94.66 ± 8.24	89.34 ± 14.19	82.13 ± 19.77	53.81 ± 17.68	65.29 ± 19.36	0.75 ± 0.80

### 3 SNPs in *GLCCI1* were associated with the change in MMEF after ICS treatment

**Fig. 2:**
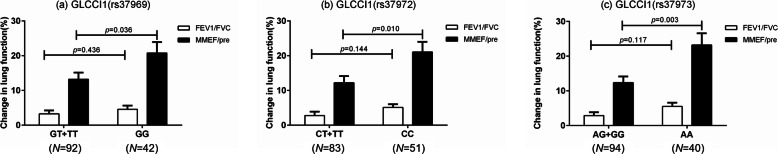
Association between *GLCCI1* SNPs and change in pulmonary function after ICS treatment for 3 months

Significant associations were identified between rs37969, rs37972, and rs37973 and the change in MMEF after 3 months of ICS treatment compared with baseline. MMEF improved by a more percentage change in subjects who were rs37969 wild-type homozygotes (GG) as compared with those who were mutant genotype (TT/GT) (20.79 ± 20.65%, 13.23 ± 18.39%, *p* = 0.036; Table 6, Fig. 2). Similar results were found in rs37972 (21.08 ± 21.03%, 12.23 ± 17.58%, *p* = 0.010; Table 6, Fig. 2) and rs37973 (23.22 ± 21.52%, 12.36 ± 17.52%, *p* = 0.003; Table 6, Fig. 2). However, this phenomenon was not repeated in rs2236647. Besides, there was also no associations between the change in FEV1/FVC and the 4 SNPs.

**Table 6:** Changes in lung function after treatment with different genotypes^a^

SNP	Biomarker	Major genotype/other genotypes	Biomarker changes in major genotype (min, max)	Biomarker changes in other genotypes (min, max)
rs37969	MMEF	GG/(GT + TT)	20.79(−19.2, 75.2)	13.23(−25.8, 64.8) ^⁎^
rs37969	PD20	GG/(GT + TT)	0.77(−0.93, 2.19)	0.44(−2.19,2.19) ^⁎^
rs37972	MMEF	CC/(CT + TT)	21.08(−19.2, 75.2)	12.23(−25.8, 64.8) ^⁎^
rs37973	MMEF	AA/(AG + GG)	23.22(−19.2, 75.2)	12.36(−25.8, 64.8) ^⁎⁎^

^a^Table only shows the SNPs that are associated with asthma susceptibility through logistic regression analysis of alleles and different genotypes

^⁎^ *p* ≤ 0.05; ^⁎⁎^ *p* ≤ 0.01

### *GLCCI1 rs37969* was associated with the change in airway hyper-responsiveness

**Fig. 3:**
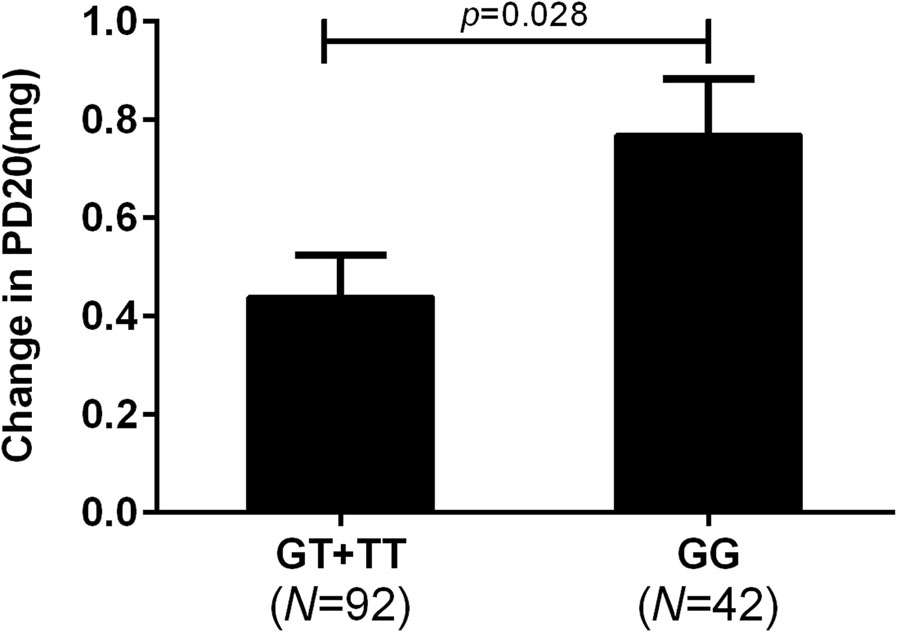
Association between the *GLCCI1* rs37969 genotype and change in PD20 after ICS treatment for 3 months

In our study, we found the mutant genotypes (TT/GT) for the *GLCCI1* rs37969 had less improvement in PD20 compared with wild-type homozygotes (GG) (0.44 ± 0.82 mg, 0.77 ± 0.74 mg; *p* = 0.028) (Fig. 3). However, we did not find the associations between the other 3 SNPs (rs2236647, rs37972, and rs37973) and the improvement in airway hyper-responsiveness.

## Discussion

Currently, the association studies between genetic variations and asthma susceptibility in population of Chinese children are still limited. To the best of our knowledge, this is the first study that confirmed the rs2236674 SNP in STIP1 gene is significantly associated with the risk of Chinese asthmatic children. We also report here that *GLCCI1* rs37969, rs37972, and rs37973 were associated with the response to ICS treatment in Chinese children with asthma.

An Arab study has indicated that the *STIP1* rs2236647 C allele can be used as an asthma marker for adult [14]. However, according to the currently reported GWAS, the *STIP1* gene has not been found to be associated with asthma sensitivity, whether in African American, Asian, Caucasian or Latino [21, 22]. In our study, C is the major allele of rs2236647 polymorphism and the frequency of wild-type homozygote (CC) in asthmatics was lower than controls. After adjusting the gender and age, we found CC homozygote children had an increased risk of asthma compared with CT/TT genotype. The previous studies were focused on adult asthma and no similar results have been reported in childhood asthma. Our finding demonstrated that CC homozygotes of *STIP1* rs2236647 polymorphism might be an asthma susceptibility marker in Chinese childhood asthma. Moreover, a white adult asthma study identified that *STIP1* rs2236647 was associated with change in lung function after ICS treatment for 4 weeks [13]. But in our study, there was no association between *STIP1* rs2236647 and the change of lung function after ICS treatment for 3 months in Chinese children. We suspected that the differences between childhood asthma and adult asthma were probably due to the age and different underlying pathophysiological basis [23, 24]. Besides, racial differences might also play important roles in these differences. However, the underlying mechanism for these differences is still unclear, and more studies are urgently needed to further explain the reasons.

In a Saudi Arabian study, 2 *GLCCI1* SNPs, (rs37972 and rs37973), were found to be unrelated to adult asthma susceptibility [25]. Similarly, we found *GLCCI1* rs37969, rs37972, and rs37973 polymorphisms were all irrelevant to the risk of childhood asthma in the current study. In 2011, *Tantisira* et al. discovered that the *GLCCI1* SNPs, (rs37972 and rs37973), was associated with change in lung function after ICS treatment in 1053 asthmatic patients [16]. Then, *GLCCI1* rs37972 and rs37973 variant genotypes were found to be related to less improvement in the FEV1 after ICS treatment for 12 weeks in Chinese patients [19]. Similar results were replicated in another Chinese study [26]. Associations also were found between *GLCCI1* rs37973 and ICS response in Japanese adult asthmatics [27]. However, a non-Hispanic white study discovered that rs37973 was not associated with the change in FEV1 after treatment with ICS [28]. Negative results were showed in a Saudi Arabian study and a recent GWAS study [25, 29]. Most of the above studies were conducted on adult asthma and there are fewer studies on these SNPs in children with asthma. In our study, we found there were no associations between *GLCCI1* polymorphisms and the improvement in FEV1/pre and FEV1/FVC after ICS treatment in childhood asthma. However, *GLCCI1* rs37969, rs37972, and rs37973 mutant genotypes were found to be associated with less improvement of the MMEF after ICS treatment. MMEF may be more sensitive than FEV1 when assessing the lung function of asthmatics [30, 31]. Therefore, our study in Chinese Han childhood asthma population support the perspective that *GLCCI1* might be considered as a predictor of ICS response to a certain extent. However, there are fewer studies on these SNPs in children with asthma compared with adults. More studies on childhood asthma are urgently needed to enrich the current theories, and studies of larger sample size and different populations are also needed to reproduce these results.

It is especially noticed that asthmatic children with *GLCCI1* rs37969 mutant genotypes have lower ICS response compared with the wild-gene homozygote in our study. Rs37969 is located in the intron region of *GLCCI1* (https://www.ncbi.nlm.nih.gov/snp/). The current data on *GLCCI1* rs37969 is extremely lacking, especially in childhood asthma. More basic experimental studies are needed to confirm whether the mutation affects the expression of GLCCI1.

There are still several limitations in this study. First, the follow-up for asthmatic children in this study was only 3 months. The follow-up period could be extended in the future. Second, the number of participants was small for a genetic study, especially in the follow-up group. Third, this study focuses on the effect of single SNPs on childhood asthma. Gene-gene interaction, epigenetics, and environment need to be considered in the future [32, 33].

## Conclusions

**Fig. 4:**
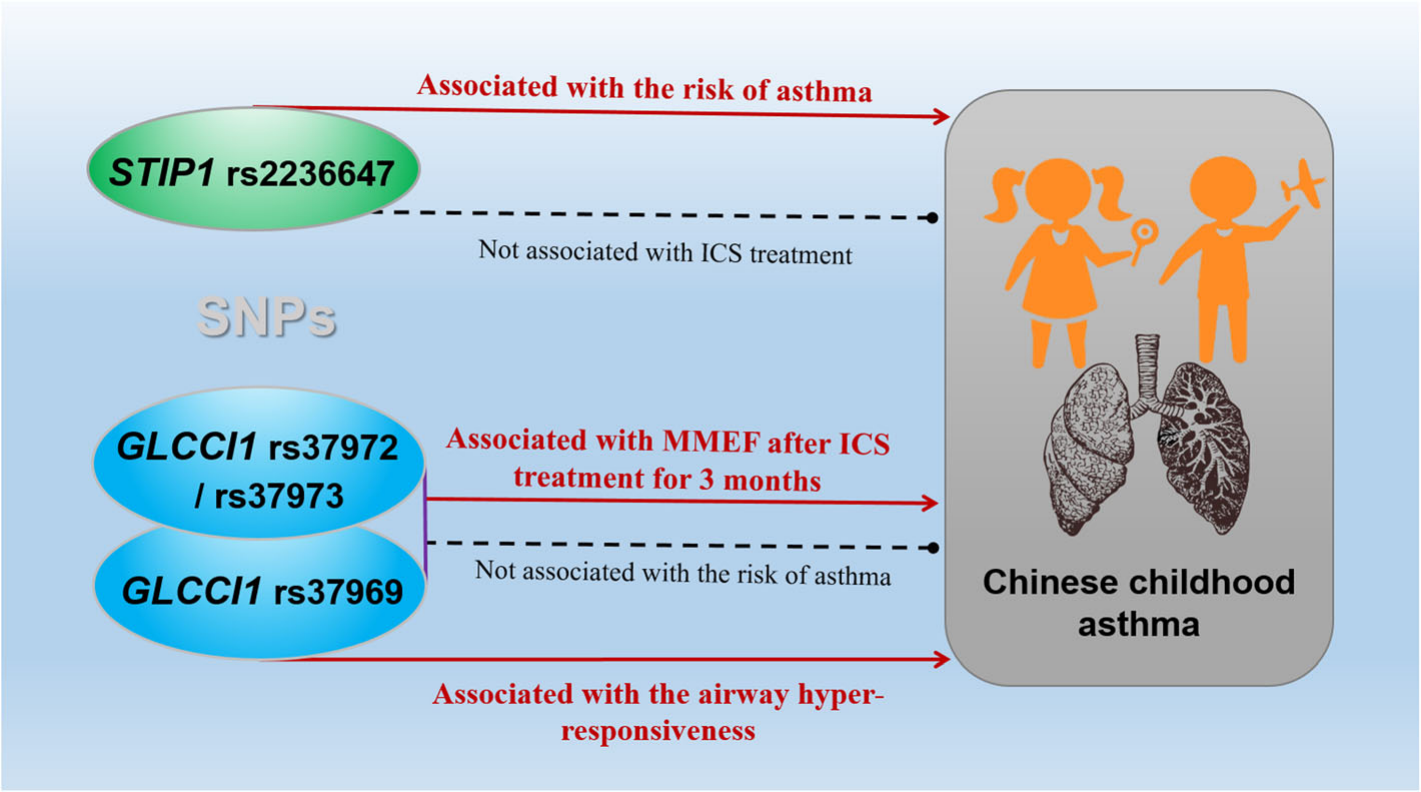
Main findings of the current study. MMEF: Maximal midexpiratory flow; ICS: Inhaled corticosteroid

In conclusion, we found significant associations between the *STIP1* rs2236647 polymorphism and the risk of childhood asthma, and *GLCCI1* SNPs are related to the improvement of lung function in Chinese Han childhood asthma patients who received ICS for 3 months. Our results indicated that *STIP1* might be considered as an asthma marker in children, while *GLCCI1* might be used to predict the ICS response in childhood asthma and Fig. 4 summarizes the main findings of the current study. It is worth mentioning that we are the first to report the function of *STIP1* rs2236647 and *GLCCI1* rs37969 in childhood asthma patients and more studies are required to repeat our findings.

## Supplementary Information

**Additional file 1: Table S1.** Primers used in genes genotyping. **Table S2.** Hardy-Weinberg equilibrium test. **Table S3.** Interaction between SNPs of our candidate genes and corticosteroid response in patients with asthma.

## Data Availability

The data generated or analyzed during this study are included in this article and its supplementary information files.

## Abbreviations

ICS: Inhaled corticosteroid
STIP1: Stress-induced phosphoprotein 1
GLCCI1: Glucocorticoid-induced transcript 1
GR: Glucocorticoid receptor
GC: Glucocorticoid
FEV1: Forced expiratory volume in 1 s
PEF: Peak expiratory flow
FEF: Forced expiratory flow
FVC: Forced vital capacity
MMEF: Maximal midexpiratory flow
PD20: Provocative dose of methacholine causing a 20% drop in FEV1

## References

[1] Soriano JB, Abajobir AA, Abate KH, Abera SF, Agrawal A, Ahmed MB (2017). Global, regional, and national deaths, prevalence, disability-adjusted life years, and years lived with disability for chronic obstructive pulmonary disease and asthma, 1990-2015: a systematic analysis for the global burden of disease study 2015. Lancet Respir Med.

[2] To T, Stanojevic S, Moores G, Gershon AS, Bateman ED, Cruz AA (2012). Global asthma prevalence in adults: findings from the cross-sectional world health survey. BMC Public Health.

[3] Asher I, Pearce N (2014). Global burden of asthma among children. Int J Tuberc Lung Dis.

[4] Price D, Fletcher M, van der Molen T (2014). Asthma control and management in 8,000 European patients: the REcognise asthma and LInk to symptoms and experience (REALISE) survey. NPJ Prim Care Respir Med.

[5] Wong GW, Kwon N, Hong JG, Hsu JY, Gunasekera KD (2013). Pediatric asthma control in Asia: phase 2 of the asthma insights and reality in Asia-Pacific (AIRIAP 2) survey. Allergy.

[6] Su N, Lin J, Chen P, Li J, Wu C, Yin K (2013). Evaluation of asthma control and patient's perception of asthma: findings and analysis of a nationwide questionnaire-based survey in China. J Asthma.

[7] Drazen JM, Silverman EK, Lee TH (2000). Heterogeneity of therapeutic responses in asthma. Br Med Bull.

[8] Palmer LJ, Silverman ES, Weiss ST, Drazen JM (2002). Pharmacogenetics of asthma. Am J Respir Crit Care Med.

[9] Garcia-Sanchez A, Isidoro-Garcia M, Garcia-Solaesa V, Sanz C, Hernandez-Hernandez L, Padron-Morales J (2015). Genome-wide association studies (GWAS) and their importance in asthma. Allergol Immunopathol (Madr).

[10] Yang IV, Lozupone CA, Schwartz DA (2017). The environment, epigenome, and asthma. J Allergy Clin Immunol.

[11] Duong-Thi-Ly H, Nguyen-Thi-Thu H, Nguyen-Hoang L, Nguyen-Thi-Bich H, Craig TJ, Duong-Quy S (2017). Effects of genetic factors to inhaled corticosteroid response in children with asthma: a literature review. J Int Med Res.

[12] Qin Q, Chen X, Feng J, Qin L, Hu C (2014). Low-intensity aerobic exercise training attenuates airway inflammation and remodeling in a rat model of steroid-resistant asthma. Chin Med J.

[13] Hawkins GA, Lazarus R, Smith RS, Tantisira KG, Meyers DA, Peters SP (2009). The glucocorticoid receptor heterocomplex gene STIP1 is associated with improved lung function in asthmatic subjects treated with inhaled corticosteroids. J Allergy Clin Immunol.

[14] Almomani BA, Al-Eitan LN, Samrah SM, Al-Quasmi MN, McKnight AJ (2017). Candidate gene analysis of asthma in a population of Arab descent: a case-control study in Jordan. Perinat Med.

[15] Izuhara Y, Matsumoto H, Kanemitsu Y, Izuhara K, Tohda Y, Horiguchi T (2014). GLCCI1 variant accelerates pulmonary function decline in patients with asthma receiving inhaled corticosteroids. Allergy.

[16] Tantisira KG, Lasky-Su J, Harada M, Murphy A, Litonjua AA, Himes BE (2011). Genomewide association between GLCCI1 and response to glucocorticoid therapy in asthma. N Engl J Med.

[17] Szalai R, Matyas P, Varszegi D, Melegh M, Magyari L, Jaromi L (2014). Admixture of beneficial and unfavourable variants of GLCCI1 and FCER2 in Roma samples can implicate different clinical response to corticosteroids. Mol Biol Rep.

[18] Vijverberg SJ, Tavendale R, Leusink M, Koenderman L, Raaijmakers JA, Postma DS (2014). Pharmacogenetic analysis of GLCCI1 in three north European pediatric asthma populations with a reported use of inhaled corticosteroids. Pharmacogenomics.

[19] Hu C, Xun Q, Li X, He R, Lu R, Zhang S (2016). GLCCI1 variation is associated with asthma susceptibility and inhaled corticosteroid response in a Chinese Han population. Arch Med Res.

[20] Salhi M, Lahmar O, Salah MO, Banic I, Binghao B, Malik W, et al. GLCCI1 and STIP1 variants are associated with asthma susceptibility and inhaled corticosteroid response in a Tunisian population. J Asthma. 2019:1–10. 10.1080/02770903.2019.1666867 31516081

[21] Hernandez-Pacheco N, Pino-Yanes M, Flores C (2019). Genomic predictors of asthma phenotypes and treatment response. Front Pediatr.

[22] Kim KW, Ober C (2019). Lessons learned from GWAS of asthma. Allergy, Asthma Immunol Res.

[23] Fuchs O, Bahmer T, Weckmann M, Dittrich AM, Schaub B, Rosler B (2018). The all age asthma cohort (ALLIANCE) - from early beginnings to chronic disease: a longitudinal cohort study. BMC Pulm Med.

[24] In SD, Song DJ, Baek HS, Shin M, Yoo Y, Kwon JW (2019). Korean childhood asthma study (KAS): a prospective, observational cohort of Korean asthmatic children. BMC Pulm Med.

[25] Al-Muhsen S, Vazquez-Tello A, Jamhawi A, Al-Dosari MS, Mahboub B, Iqbal N (2015). Rs37972 and rs37973 single-nucleotide polymorphisms in the glucocorticoid-inducible 1 gene are not associated with asthma risk in a Saudi Arabian population. J Asthma.

[26] Xu Y, Wu H, Wu X, Xu Y, Zhao J, Xie J (2017). GLCCI1 rs37973: a potential genetic predictor of therapeutic response to inhaled corticosteroids in Chinese asthma patients. Medicine (Baltimore).

[27] Rijavec M, Zavbi M, Lopert A, Flezar M, Korosec P (2018). GLCCI1 polymorphism rs37973 and response to treatment of asthma with inhaled corticosteroids. J Investig Allergol Clin Immunol.

[28] Hosking L, Bleecker E, Ghosh S, Yeo A, Jacques L, Mosteller M (2014). GLCCI1 rs37973 does not influence treatment response to inhaled corticosteroids in white subjects with asthma. J Allergy Clin Immunol.

[29] Mosteller M, Hosking L, Murphy K, Shen J, Song K, Nelson M (2017). No evidence of large genetic effects on steroid response in asthma patients. J Allergy Clin Immunol.

[30] Ciprandi G, Cirillo I, Pasotti F, Ricciardolo FL (2013). FEF25-75: a marker for small airways and asthma control. Ann Allergy Asthma Immunol.

[31] Simon MR, Chinchilli VM, Phillips BR, Sorkness CA, Lemanske RJ, Szefler SJ (2010). Forced expiratory flow between 25 and 75% of vital capacity and FEV1/forced vital capacity ratio in relation to clinical and physiological parameters in asthmatic children with normal FEV1 values. J Allergy Clin Immunol.

[32] Deng Q, Lu C, Li Y, Sundell J, Dan N (2016). Exposure to outdoor air pollution during trimesters of pregnancy and childhood asthma, allergic rhinitis, and eczema. Environ Res.

[33] Deng Q, Lu C, Norback D, Bornehag CG, Zhang Y, Liu W (2015). Early life exposure to ambient air pollution and childhood asthma in China. Environ Res.

