# Supplementary material for: Effects of STIP1 and GLCCI1 polymorphisms on the risk of childhood asthma and inhaled corticosteroid response in Chinese asthmatic children
Source: BMC Pulm Med. 2020 Nov 18;20:303. doi: 10.1186/s12890-020-01332-2 (PMC7677774; doi:10.1186/s12890-020-01332-2)
Supplement: Supplementary file 1 — Additional file 1: Table S1. Primers used in genes genotyping. Table S2. Hardy-Weinberg equilibrium test. Table S3. Interaction between SNPs of our candidate genes and corticosteroid response in patients with asthma. [file 12890_2020_1332_MOESM1_ESM.docx]

**Additional file 1**

**Table S1. Primers used in genes genotyping**

| **Gene** | **SNPs** | **Primers** |
| --- | --- | --- |
| *STIP1* | rs2236647 | Forward: ACGTTGGATGCAAGTTCTCCTGAAGGCCTC |
|  |  | Reverse: ACGTTGGATGCAGAGCAAGCAAAGATGAAG |
| *GLCCI1* | rs37969 | Forward: ACGTTGGATGTTTCACCCAGACAGACTGGC |
|  |  | Reverse: ACGTTGGATGATGTACCCCACAGCTTTGAG |
|  | rs37972 | Forward: ACGTTGGATGCACGAGCAGACCAATTTGAC |
|  |  | Reverse: ACGTTGGATGCGGGAGCAATTAATGTAAGG |
|  | rs37973 | Forward: ACGTTGGATGCAAATGTTAGGCTGTAAGAG |
|  |  | Reverse: ACGTTGGATGAAGAACTTCTGGTGATCAGG |

**Table S2. Hardy-Weinberg equilibrium test.**

| **Gene** | **SNP** | **Minor allele** | **All**  **(n=413)** | **Cases**  **(n=263)** | **Controls**  **(n=150)** |
| --- | --- | --- | --- | --- | --- |
| *STIP1* | rs2236647 | C | 0.536 | 0.285 | 0.622 |
| *GLCCI1* | rs37969 | G | 0.843 | 1 | 0.869 |
|  | rs37972 | C | 0.361 | 0.445 | 0.616 |
|  | rs37973 | A | 0.621 | 0.803 | 0.626 |

**Table S3. Interaction between SNPs of our candidate genes and corticosteroid response in patients with asthma.**

| Gene | Population | Result | References |
| --- | --- | --- | --- |
| *STIP1* | 382 white asthmatic adults | STIP1 genetic variations might play a role in regulating corticosteroid response in asthmatic subjects with reduced lung function. | [13] |
|  | 230 asthmatic adults; Arab | rs2236647 C allele can be used as an asthma marker | [14] |
| *GLCCI1* | 271 asthmatic adults; Saudi Arabia | rs37972 and rs37973 were unrelated to adult asthma susceptibility | [25] |
|  | FBAT screening cohort in the CAMP clinical trial population | The wild-type allele of rs37973 was associated with significant decrements in FEV1 | [16] |
|  | 182 asthmatic adults; China | The rare alleles of rs37972 and rs37973 were correlated with less improvement in FEV1 after fluticasone treatment | [19] |
|  | 230 asthmatic adults; Tunisian | The T allele rs37972 and G allele rs37973 were correlated with a strong risk for developing severe asthma; the G allele of rs37973 was associated with worse response to ICS | [20] |
|  | 224 asthmatic adults; Japan | The GG genotype of GLCCI1 rs37973, is a risk factor of pulmonary function decline in patients with asthma receiving long-term ICS treatment. | [27] |
|  | 1791 asthmatic children; Northern Europe | Variation in GLCCI1 rs37972 genotype does not seem to affect ICS efficacy | [28] |
